# Stability of multiple alignments and phylogenetic trees: an analysis of ABC-transporter proteins family

**DOI:** 10.1186/1748-7188-3-15

**Published:** 2008-11-06

**Authors:** Holger Wagner, Burkhard Morgenstern, Andreas Dress

**Affiliations:** 1 FSPM, Faculty of Mathematics, Bielefeld University, Postfach 100131, D-33501 Bielefeld, Germany; 2 Institute of Microbiology and Genetics, University of Göttingen, Goldschmidtstr. 1, D-37077 Göttingen, Germany; 3 CAS-MPG Partner Institute for Computational Biology, 320 Yue Yang Road, 200031 Shanghai, PR China; 4 Max Planck Institute for Mathematics in the Sciences, Inselstrasse 22 – 26, D-04103 Leipzig, Germany

## Abstract

**Background:**

Sequence-based phylogeny reconstruction is a fundamental task in Bioinformatics. Practically all methods for phylogeny reconstruction are based on multiple alignments. The quality and stability of the underlying alignments is therefore crucial for phylogenetic analysis.

**Results:**

In this short report, we investigate alignments and alignment-based phylogenies constructed for a set of 22 ABC transporters using CLUSTAL W and DIALIGN. Comparing the 22 "one-out phylogenies" one can obtain for this sequence set, some intrinsic phylogenetic instability is observed — even if attention is restricted to branches with high bootstrapping frequencies, the so-called safe branches. We show that this instability is caused by the fact that both, CLUSTAL W as well as DIALIGN, apparently get "confused" by sequence repeats in some of the ABC-transporter. To deal with such problems, two new DIALIGN options are introduced that prove helpful in our context, the "exclude-fragment" (or "xfr") and the "self-comparison" (or "sc") option.

**Conclusion:**

"One-out strategies", known to be a useful tool for testing the stability of all sorts of data-analysis procedures, can successfully be used also in testing alignment stability. In case instabilities are observed, the sequences under consideration should be carefully checked for putative causes. In case one suspects sequence repeats to be the cause, the new "sc" option can be used to detect such repeats, and the "xfr" option can help to resolve the resulting problems.

## 1 Introduction

For more than three decades, sequence-based computer programs for phylogenetic reconstruction are routinely used for sequence-data analysis, see [10,34,11] for reviews of various popular methods. They are crucial tools not only for understanding gene and species evolution, but also for analyzing the structure and function of proteins. A good example is the family of ABC-transporter proteins that became a topic of active research during the last few years (ABC stands for ATP-Binding Casette). At present, thousands of different ABC transporters are known; for the bacterium *Sinorhizobium meliloti *alone, around 150 ABC-transporter proteins have been identified [2,4]. Thus, the sheer size of the ABC-transporter family makes it desirable to reduce experimental costs by using *in silico *methods for structural and functional annotation. In view of some recent articles in which a close correspondence between the phylogeny and physiological function of these proteins was demonstrated (cf. [5,15,18,19,30]), phylogenetic analysis may apparently be of some use in this context: Putative functions of unknown ABC transporters can be delineated from their position within the family tree. However, this perspective of using tree-reconstruction programs for *in silico *annotation makes it indispensable to thoroughly check their current reliability, as well as that of the multi-alignment programs on which such reconstruction programs are based.

**Figure 1:**
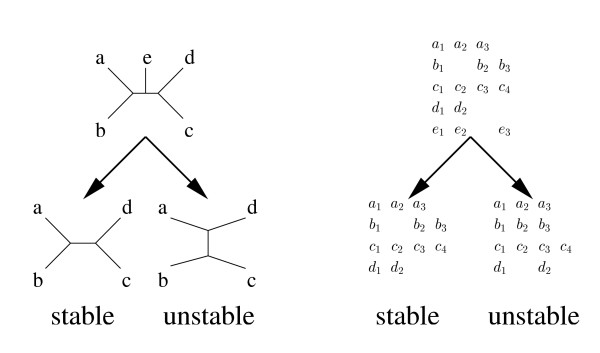
**Stability versus instability of molecular phylogenies (left) and alignments (right): A tree-reconstruction program produces a stable phylogeny if the phylogeny derived for a smaller sequence set coincides with the sub-phylogeny induced, for this smaller set, from the 'complete' phylogeny.** Analogously, a multi-alignment program produces a stable alignment if the alignments of smaller sequence sets show the same columns as the corresponding subalignment of the 'complete' alignment.

Herein, we study the reliability of two standard multi-alignment programs, CLUSTALW and DIALIGN, by applying them to ABC transporters. We apply the "one-out strategy" to analyze the stability of the alignments constructed by these two methods, i.e., by removing – one by one – individual sequences from the input sequence set: Ideally, removing sequences from a sequence set should not affect the alignment of the remaining sequences; their alignment should agree with the subalignment derived for them by restricting the alignment constructed for the complete sequence set (Fig. 1). But, as we will demonstrate, this stability requirement is often not fulfilled, and the resulting instabilities of alignments may even cause – and can be detected by – instabilities of the branching structure of the phylogenetic trees constructed from the alignments.

The most popular tool to test the reliability of a tree topology is the bootstrapping method. It works as follows [9,6]: for a fixed alignment of sequences, a collection of new alignments is generated by deleting some columns from the alignment at random and replacing them by other columns from that alignment, also picked at random. The tree-building algorithm is then applied to the resulting collection of new alignments. The relative frequency with which a chosen branch appears, its *bootstrapping frequency*, is then taken as a measure of the confidence one can have in this branch – or, more precisely, in the phylogenetic relevance of the corresponding *split *of the underlying sequence set induced by this branch. Hence, bootstrapping can be used to assess the reliability of a tree's branches derived from a fixed alignment and to identify its *safe *branches that we define as branches with a bootstrapping frequency above a certain threshold, but it does not reflect the reliability of the alignment itself. For this paper, we used a threshold value of 5/6.

In this study, only the safe branches of a tree topology will be considered when comparing trees resulting from distinct alignments, and we will confirm that, as expected, instabilities concerning these branches indicate instabilities of the underlying alignments.

Furthermore, we show that a new option that has been added in the context of our investigations to the DIALIGN software can lead to considerably improved alignments. This option, called the *exclude-fragment *or, for short, the *xfr option*, allows the user to exclude certain pairs of sites, or pairs of segments, from being aligned to each other and, this way, to introduce expert knowledge into the alignment process as well as to explore interactively various alignment alternatives. In this regard, the *xfr option *is sort of "dual" to the recently introduced *anchored-alignment *option where the program is *required *to align certain user-specified parts of the sequences to each other – provided the requirements are consistent and there is at least some degree of similarity between the specified regions [3,27]. In this context, we also look at the influence of the *guide tree *used by CLUSTAL W for constructing CLUSTAL W alignments.

As a by-product of our study, we found that the numerical values of the scores of multiple alignments produced by DIALIGN are sometimes noticeably below the scores of other multiple alignments that DIALIGN can be tricked to find for the same sequence set using the *xfr *option. This observation, while perhaps disturbing, but not of immediate relevance for the *user*, is apparently crucial for further *development *of this tool. Indeed, every multi-alignment approach has to address two major problems: (a) a procedure has to be defined that, in principle, associates a *score *to *every *possible alignment of any given sequence set and (b) an *optimization algorithm *has to be designed that finds optimal (or near-optimal) alignments relative to that scoring procedure. Our study shows that DIALIGN can sometimes fail by some margin to find the maximum score indicating that improvements of its optimization procedures could lead to considerable improvement of the quality and, in particular, to "stabilization" of the alignments constructed by DIALIGN. Note that this result is not self-evident: According to the two parts of the alignment problem, there can be two reasons for mis-alignments as detected by instabilities: (i) the employed scoring function may itself be "unstable" (in which case it can definitely not always be in accordance with biology), and (ii) the optimization algorithm produces alignments with scores significantly below the mathematical optimum. Our study shows that the latter problem actually plays a non-negligible role. Thus, it makes sense to develop alternative optimisation algorithms for the fragment-based alignment approach [17,33,32].

One reason for the instabilities of the alignments constructed for the set of ABC transporters under investigation is the repetitive structure of three of its sequences. In a last step, we will therefore decompose these sequences into non-repetitive parts by applying another new option of DIALIGN. For the resulting new sequence set, we will also compute alignments and phylogenetic trees. Remarkably, the DIALIGN algorithm produces perfectly stable alignments and phylogenies for this new data set.

The paper is organized as follows: first, we shortly review the two multi-alignment programs CLUSTAL W [35] and DIALIGN [24,23] that we are using for our analysis. We discuss some of their weak points that proved to be relevant in our context, and we indicate possible ways of overcoming them by incorporating expert knowledge. Next, we apply CLUSTAL W and DIALIGN to a set of 22 ABC transporter sequences and demonstrate that both algorithms lead to highly unstable phylogenetic trees. We show that this instability is due to instabilities of the underlying alignment procedures that are, in turn, caused by sequence repeats. Following these observations, we finally demonstrate that biologically meaningful alignments can be produced by both programs if additional information regarding these repeats or other forms of "expert knowledge" are incorporated into the alignment procedure.

## 2 Alignment procedures

In our study, we investigated CLUSTAL W since it is still the most popular software propgram for multi-alignment. It is a classical implementation of the *progressive *alignment method. That is, it reduces the computational costs of constructing a multiple alignment by reducing this task to a sequence of pairwise alignments of *profiles *representing previously calculated multiple alignments of subsets of the original sequence set. These pairwise alignments are calculated according to the classical Needleman-Wunsch algorithm [28] using a scoring scheme that sets rewards for pairs of matching residues against penalties imposed for gaps. Nowadays, there are more sophisticated progressive alignment methods available, for example MAFFT [14,13] or MUSCLE [8,7]. Investigating the behaviour of these program in detail would be beyond the scope of the present study. Thus, we restrict ourselves to CLUSTAL as a prototype of the progressive alignment methods.

A crucial question is, of course, to decide in which order the pairwise alignments are computed in a progressive approach. With default options, CLUSTALW calculates, in a first step, all $\left(\begin{array}{c} n\\ 2 \end{array}\right)$ pairwise alignments for an input set of *n *sequences, and constructs a guide tree for the input sequences based on their pairwise similarity scores. To this end, it uses the neighbor-joining algorithm [29]. The "progressive" alignment procedure, i.e., the successive alignment of sequences and/or profiles, is then carried out according to the branching pattern of this guide tree. While this method is computationally efficient, a major point of concern is that the resulting multiple alignments – and, therefore, any phylogenetic conclusions derived from them – strongly depend on the guide tree. Clearly, any instability of the guide tree will be reflected by corresponding instabilities of the resulting alignments – in particular in the *slow-and-accurate *mode in which also the score function itself depends on that guide tree. Generally, CLUSTAL W produces multiple alignments of high quality if the sequences are *globally *related and the degree of over-all similarity between them is sufficiently strong [36,16].

DIALIGN uses a completely different approach. The similarity between sequences is not measured by summing up, over all sites, properly specified individual similarity scores, but rather by comparing whole sequence segments of equal length with each other. Alignments are composed from gap-free segment pairs, so-called *fragment alignments *or just *fragments*. Every possible fragment is given a score based on the probability of its random occurrence [20], and the algorithm tries to find a *consistent *collection of fragments with maximal total score; consistency here means the combinatorial requirement that the selected fragments are required to fit into one single output alignment and do not 'cross' each other. Gaps are not penalized in this approach – and there is, in consequence, no worrying about how to quantify gap penalties.

For pairwise alignment, the "best" chain of fragments is found using an exact recursive optimization algorithm [21,22]. For multi-alignments, all optimal pairwise alignments are constructed in a first step. Taking account of their individual score, fragments occurring in these pairwise alignments are then incorporated one-by-one into a growing multiple alignment in a greedy fashion, provided they are consistent with the fragments that were included previously. The nature of this approach can, of course, lead to mis-alignments, in particular for sequences that contain repeats. A single wrong fragment accepted at an early stage of the greedy procedure can subsequently prevent many correct fragments from being included into the alignment, thereby leading to an output alignment of lower over-all quality and to instability. The order in which the fragments from the optimal pairwise alignment are checked for consistency and included into the multiple alignment is therefore crucial for the resulting multiple alignment – somehow in analogy to (but slightly less worrying than) the problems related to the choice of the guide tree used by CLUSTAL W.

Note that CLUSTAL W and DIALIGN reflect very different philosophies regarding the definition of scores: DIALIGN uses a fixed scoring procedure whereas the scoring procedures used by CLUSTAL W depends -in the slow-and-accurate mode applied in our studies – on the guide tree used for the computation of the alignment. Thus, to compare two alignments of the same sequence set, one can compare their score if both are DIALIGN alignments whereas this would not make sense if both are CLUSTAL W alignments (and even less if one is a DIALIGN and the other one a CLUSTAL W alignment).

Two recently implemented options of DIALIGN were used in this study:

• One way of influencing the output alignment is to *prevent *certain pairs of sequence sites, or whole gap-free sequence segments, from being aligned with each other; we implemented this *exclude-fragment *(or *xfr*) option to improve the alignment of the ABC transporters in the present study. The *xfr *option can be used to prevent early inclusion of misleading fragments in the greedy alignment procedure outlined above, but also to explore interactively various alignment alternatives.

• The *self-comparison *(or *sc*) option was implemented to search for repeats in a given sequence. To this end, a sequence is aligned with itself in such a way that any single sequence entry can be aligned to any other entry except itself.

## 3 Sequence data and definitions

### 3.1 Sequence data

In this study, all investigations of ABC transporters – or, for short, ABCs – are based on the ATPase-domains of ABC transporters. Their primary structure is characterized by three highly conserved sequence motifs: two Walker motifs called **A **and **B **that represent the ATP-decomposing domains whereas the physiological function of a third motif, the so-called signature sequence **S**, is still unknown. In [31], these subsequences are characterized as follows:

**A **: *G x x G x G K *[*S*, *T*], **S **: *L S G G Q *[*Q*, *R*, *K*] *R*, **B **: *h h h h D*,

where *x *stands for an arbitrary, and *h *for an arbitrary hydrophobic, amino acid. The order of these three motifs is – at least for the sequences considered in this study -

- **A **– **S **– **B **-

(from N- to C-terminus), with dashes representing arbitrary amino-acid sequences. Some of the ABCs comprise two sets of these motifs, so their structure is

- **A**_1 _– **S**_1 _– **B**_1 _– **A**_2 _– **S**_2 _– **B**_2 _-

More specifically, we will consider here a collection of 22 amino-acid sequences, i.e., the complete sequences of the following proteins: tr| Q9RK11| (*S*1), sp| P32010| (*S*2), sp| P23199| (*S*3), sp| P08720| (*S*4), sp| P31220| (*S*5), sp| P25885| (*S*6), sp| P31134| (*S*7), sp| P26905| (*S*8), sp| P30750| (*S*9), sp| P15031| (*S*10), sp| P07109| (*S*11), sp| P09833| (*S*12), sp| P16678| (*S*13), sp| P30963| (*S*14), sp| P23888| (*S*15), sp| P29018| (*S*16), sp| P27299| (*S*17), sp| P33116| (*S*18), sp| P33951| (*S*19), sm| b20111| (*S*20), sm| b21260| (*S*21), sm| b20141| (*S*22). Here, tr, sp, and sm denote entries in TrEMBL, Swissprot , and in , respectively.

### 3.2 Some Definitions

Let *X *denote the set of all taxa under consideration, and let *T *denote a phylogenetic tree – or, for short, a *phylogeny *– for *X*. Then, each branch *b *of this tree induces a *split S*(*b*) of *X*, i.e., a bipartition of *X *into two disjoint non-empty subsets of *X *whose union is all of *X *while *S*(*b*) ≠ *S*(*b'*) holds for any two distinct branches *b*, *b' *of *T *(cf. e.g. [1]). Thus, each phylogeny *T *with *N *branches *b*_1_, ..., *b*_*N *_gives rise to a collection Σ(*T*): {S(*b*_*i*_) : 1 ≤ *i *≤ *N*} of *N *distinct splits of *X *that, in turn, are well known to determine *T *(up to canonical isomorphism). Accordingly, if bootstrapping frequencies *f*(*b*) have been computed for every branch *b *of *T*, the set of all safe branches (i.e., of all branches *b *with a bootstrapping frequencies *f*(*b*) at least 5/6) can be identified with the set of splits Σ _*safe*_(*T*): {*S*(*b*_*i*_) : 1 ≤ *i *≤ *N*, *f *(*b*_*i*_) ≥ 5/6}.

Two phylogenies *T*_1 _and *T*_2 _of *X *are said to be *essentially concordant *if each one "contains" the safe branches of the other, i.e., if

(3.1) Σ_*safe*_(*T*_1_) ⊆ *S*(*T*_2_) and Σ_*safe*_(*T*_2_) ⊆ *S*(*T*_1_)

holds. Otherwise, *T*_1 _and *T*_2 _are called *definitively distinct*.

Let us now assume that we are given

• a collection *X *of (phylogenetically related) sequences and

• an algorithm **Alg **for computing phylogenies from sequences.

For each subset $\tilde{X}$ of *X*, let **Alg**($\tilde{X}$) denote the phylogeny computed for $\tilde{X}$ using the algorithm **Alg**. Then, **Alg **is said to produce a *stable *phylogeny for *X *if

$$\Sigma_{safe}(Alg(\tilde{X}))\subseteq \tilde{X}\cap \Sigma (Alg(X))\text{ and }\tilde{X}\cap \Sigma_{safe}(Alg(X))\subseteq \Sigma (Alg(\tilde{X}))$$

holds for all subsets $\tilde{X}$ of *X *of co-cardinality 1 (i.e., with#(*X *- $\tilde{X}$) = 1) where, for every subset $\tilde{X}$ of *X *and every collection of Σ of splits of *X*, we denote by $\tilde{X}$ ∩ Σ the corresponding collection of splits of $\tilde{X}$ defined by

$$\tilde{X}\cap \Sigma :=\{\{Y\cap \tilde{X},Z\cap \tilde{X}\}:\{Y,Z\}\in \Sigma ,Y\cap \tilde{X},Z\cap \tilde{X}\neq \emptyset \}.$$

## 4 Results

### 4.1 Molecular phylogenies

**Figure 2:**
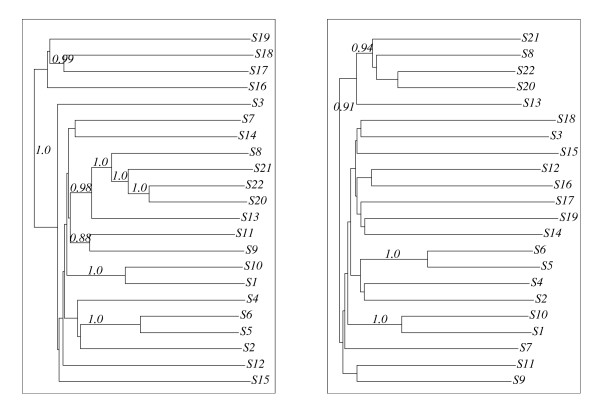
**Two tree topologies: The tree *T*_1 _(left) representing – up to unsafe branches – the "Topology I trees" was computed for our sequence family  using CLUSTALW, and *T*_2 _(right) representing – up to unsafe branches – the "Topology II trees" was computed using DIALIGN.** The bootstrapping frequencies are shown for the safe branches of each tree. Note that the safe branches in *T*_1 _separating{*S*17, *S*18}, {*S*16, *S*17, *S*18, *S*19}, and {*S*20, *S*21, *S*22}, respectively, from the sequences disapper in *T*_2_.

The sequence family ⅅ as aligned using CLUSTAL Wand DIALIGN. DIALIGN was used with default parameters and CLUSTAL W was run with the slow-and-accurate option. Subsequently, phylogenies were computed from the alignments using the neighbor-joining algorithm with the bootstrapping option of CLUSTAL W (1000 trials, seed of 111). This resulted in two definitively distinct phylogenies depicted in Fig. 2 (*T*_1 _for CLUSTAL W and *T*_2 _for DIALIGN).

**Figure 3:**
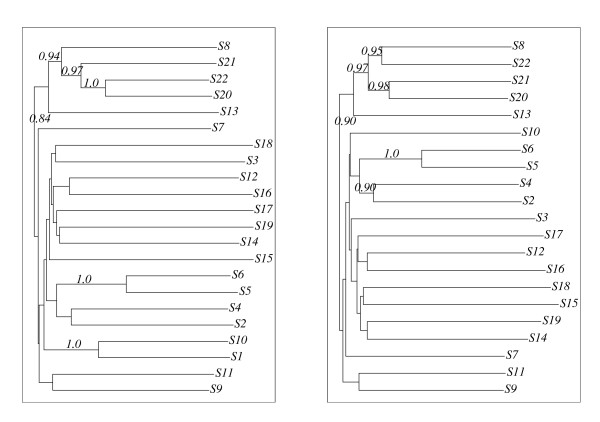
**Representatives of "Topology III trees" (left) and "Topology IV trees" (right): Both are derived by DIALIGN. **The left phylogeny is computed for  using the xfr option (cf. 2 and 4.3) whereas the right one resulted from applying DIALIGN to _1 _:{*S*2, ..., *S*22}.

To investigate the stability of these phylogenies, we also computed phylogenies for the 22 "reduced" sequence families ⅅ_*i *_consisting of all sequences in ⅅ except the sequence *Si *(*i *= 1, ..., 22) in the same fashion. Each of these phylogenies was essentially concordant with (the restriction to the respective sequence sets of) one of the trees shown in Fig. 2 and 3. More specifically, the CLUSTAL W alignments of ⅅ_8_, ⅅ_10_, and ⅅ_22 _led to Topology III trees (cf. Fig. 3) whereas the CLUSTAL W alignments of all other reduced sequence families led to Topology I trees. The DIALIGN alignments resulted in Topology III trees for ⅅ_5 _and ⅅ_22_, Topology IV trees for ⅅ_1_, ⅅ_8_, ⅅ_20_, and ⅅ_21_, and Topology II trees for all other reduced sequence families. Hence, CLUSTAL W and DIALIGN produced not only definitively distinct, but also highly unstable phylogenies for our sequence families.

### 4.2 Underlying alignments

**Figure 4:**
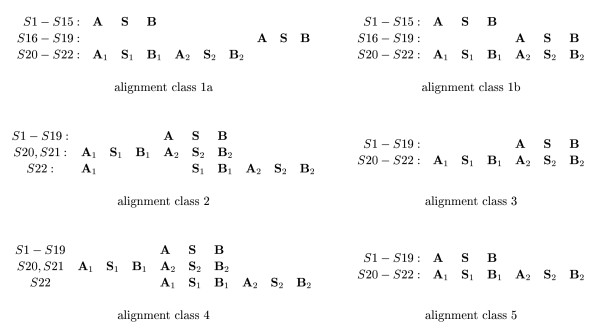
**For most alignment algorithms, sequence duplications can lead to serious mis-alignments and to instabilities under minor modifications of the input sequence sets.** For our original sequence set  = {*S*1, ..., *S*22} and the 'reduced' sequence sets _*i *_:= \{*Si*}, we obtained six classes of multi-alignments. Here, class 3 and 5 seem to be the only types of alignments that are biologically meaningful. Alignments of class 5 were obtained only once, namely by DIALIGN using the xfr option as described in 2 and 4.3. We used this option to prevent the program from aligning the motifs A_2_, *S*_2 _and B_2 _in the sequences S20 – S22 to the motifs A, S and B in the remaining sequences.

To investigate the reasons for the occurrence of these rather distinct tree topologies, we analysed the underlying alignments. In doing so, we classified these alignments by the relative positions of the motifs **A**, **S**, and **B**. This classification led to the 5 alignment classes 1a, 1b, 2, 3, and 4 shown in Fig. 4 one of which, class 1a, was observed only once, *viz*., for ⅅ_14_. Among these alignments, alignments in class 3 look biologically most meaningful since it implies only one duplication event. By contrast, alignment in class 1a, 1b, 2 and 4 would require at least two independent duplication or deletion events. A meaningful alternative, namely alignments of class 5, did occur only after applying the *xfr *option of DIALIGN for excluding class-3 type alignments.

Next, we found that alignments of class 1a and 1b always led to Topology I trees. Analogously, alignments in the classes 2, 3, and 4 always yielded Topology II, III, and IV trees, respectively. This showed clearly that the instabilities and inconsistencies of the safe branches can be traced back to the underlying alignments.

### 4.3 Modified alignments

In this subsection, we discuss which of the 5 alignment classes have the best DIALIGN score. To compute DIALIGN scores for all alignment classes, the *xfr *option described in **2 **was used: We excluded the possibility of aligning the subwords **S**_1 _and **B**_1 _of *S*22 with subwords of any of the other sequences aligned with these subwords in the class-2 or class-4 alignments. This led, for all 23 sequence sets ⅅ, ⅅ_1_, ..., ⅅ_22_, to alignments of class 3. Moreover, the scores of these class-3 alignments (7933–9367) were between 140 and 559 "DIALIGN units" higher than the scores of the corresponding class-2 or class-4 alignments. In view of the fact that the DIALIGN scores are computed in terms of the logarithm of probabilities [24], these differences are quite substantial.

Analogously, the *xfr *option of DIALIGN was used to exclude all fragments of class-2 and class-4 alignments that involve the subwords **S**_2 _or **B**_2 _of the sequences *S*20 or *S*21. As expected, this led to an alignment of class 5 (cf. Fig. 4) whose score 9230 was 137 "DIALIGN units" smaller than the score of the corresponding class-3 alignment. So, class-5 alignments appear to represent suboptimal alignments with respect to the DIALIGN score.

To investigate the influence of the guide tree on CLUSTAL W alignments, we proceeded as follows: As mentioned above, the CLUSTAL W alignment of ⅅ_10 _led to an alignment of class 3. In the guide tree of this alignment, we systematically replaced, quite crudely, *Si *by *S*10 and used this new guide tree for a CLUSTAL W alignment of ⅅ_*i*_.

**Figure 5:**
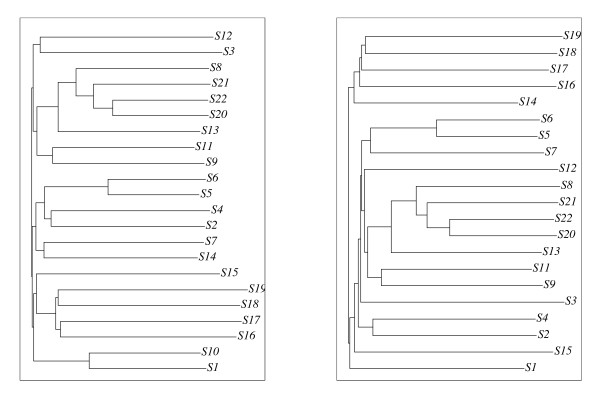
**The guide trees of the CLUSTAL W alignments of  (left) and of _10 _(right).** The left guide tree resulted also from an alignment of class (1b), the right one also from an alignment of class 3. Note that both exhibit a branch that separates the sequences S16–S19 from the remaining sequences.

In each case, this resulted in an alignment of class 3. This is quite surprising since the two guide trees leading to these rather different alignments, do not look too different. In Fig. 5, 10 out of 20 internal nodes are identical for both trees i.e. the corresponding sub-clades coincide precisely. In particular, all guide trees include a branch with taxa *S*16–*S*19, i.e., the problematic sequences in the alignments of class 1a and 1b (cf. Fig. 4). Thus, our investigations demonstrate that small modifications of the guide tree can change the alignment significantly and that inappropriate guide trees can result in biologically unacceptable alignments. It is worth noting that all alignments of class 3 or 5 led to Topology III trees.

### 4.4 Decomposition in N- and C-termini

Our analyses showed that one reason for the observed instabilities is the repetitive structure of the last three sequences S20–22. Accordingly, we constructed two sequences out of each of these three sequences, each new sequence containing a single -**A **– **S **– **B**- motif. To this end, we aligned each of these three sequences to itself by applying the sc option of DIALIGN to identify, and later to excise either one of, their repeats. This way, we created a new sequence family ⅅ*' *consisting of sequences *S*1 – *S*19 together with six new sequences *S*20*N*, *S*21*N*, *S*22*N*, *S*20*C*, *S*21*C*, and *S*22*C *containing either the left-hand, but not the right-hand, or the right-hand, but not the left-hand, repeats of the respective sequences.

**Figure 6:**
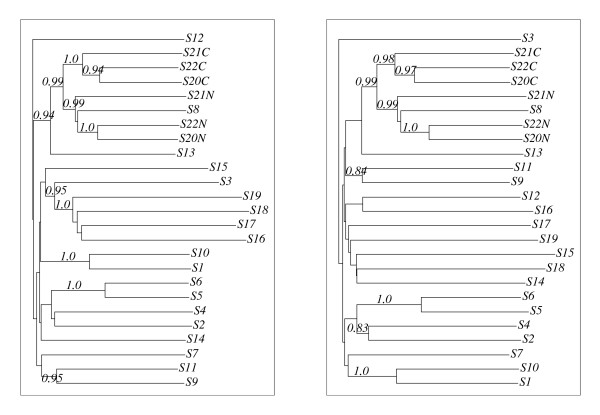
**Phylogenies for the sequence set *'*.** On the left (right) hand side, one finds the phylogeny derived from the CLUSTAL W (DIALIGN) alignment.

We investigated the phylogenies and alignments for the new sequence set in the same fashion as for the original sequence family. Fig. 6 shows the phylogenies derived from the CLUSTAL W resp. DIALIGN alignment. While the latter produced a perfectly stable phylogeny, CLUSTAL W produced only an "almost stable" phylogeny – only the branch corresponding to the split of ⅅ*' *into {*S*3, *S*16, *S*17, *S*18, *S*19} and its complement was found to be safe, yet it did not occur in any tree constructed for a proper subset of ⅅ*'*.

**Figure 7:**
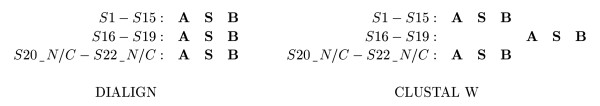
**Alignments for *'*.** The DIALIGN alignment (left) was found to be stable. The other alignment is only one example of a variety of different alignments obtained for this sequence set and its subsets.

The DIALIGN alignment was also shown to be stable (see Fig. 7) whereas a variety of different alignment classes showed up in the CLUSTAL W alignments, one example being also shown in Fig. 7. But there was one common feature of the CLUSTAL W alignments: The **A**, **S**, and **B **motifs of *S*16 – *S*19 were never aligned to the corresponding motifs of the other sequences.

Note that the restrictions of all CLUSTAL W and DIALIGN alignments to the sequences *S*1–*S*19 coincide, respectively, with the corresponding restrictions of the alignments of class 1 or class 3 (see Fig. 4). Thus, it is no surprise that the restrictions of the trees of ⅅ*' *to the taxa *S*1 to *S*19 also coincide with the corresponding restrictions of the Topology I (CLUSTAL W) and Topology III (DIALIGN) trees shown in Fig. 2 and 3.

This demonstrates that the two different approaches towards improving the DIALIGN alignment, namely the exclusion of fragments and the decomposition of the repetitive sequences in N- and C-termini, do not lead to contradictory trees.

Furthermore, these analyses of the CLUSTAL W alignments and the trees derived from them explain in particular the stability of the branch containing the four taxa *S*16 to *S*19. And they show that not all the problems concerning the CLUSTALW alignments of the ABCs are caused by repeats. Additionally, we analyzed the alignments and the trees of the sequence set ⅅ*''*: = {*S*1, ..., *S*19}: Again DIALIGN produced a stable alignment and a stable phylogeny that coincided with the restriction of a class-3 alignment and a Topology III tree to this subset, respectively, while the alignment as well as the phylogeny produced by CLUSTAL W still turned out to be unstable: The tree produced by CLUSTAL W for ⅅ*'' *coincides with the restriction of the tree derived for ⅅ*'*. But the sequence set consisting of *S*1–*S*6 and *S*8–*S*19, for example, leads to an alignment of class 3 and a Topology III tree.

### 4.5 Relation between branches and function

So far, we emphasized differences between tree topologies and the problems concerning their stability. In this section, we discuss which branches all our trees have in common and whether they are related to the physiological functions of the ABCs.

All six tree topologies separate the following sequence clusters from the remaining sequences (often by safe branches): {*S*1, *S*10}, {*S*2, *S*4}, {*S*5, *S*6}, {*S*9, *S*11}, {*S*8, *S*13, *S*20, *S*21, *S*22}, {*S*8, *S*20, *S*21, *S*22}, {*S*20, *S*22}.

Furthermore, replacing each of the sequences *S*20, *S*21, *S*22 by the corresponding N- and C-terminal sequences *S*20*N*, *S*21*N*, *S*22*N *and *S*20*C*, *S*21*C*, *S*22*C*, the same holds for the two trees constructed for ⅅ*' *which, in addition, separate {*S*8, *S*20*N*, *S*21*N*, *S*22*N*} suggesting a a slower evolution of the N-terminal motifs of the sequences *S*20, *S*21, *S*22 compared with the evolution of the C-terminal motifs of these sequences.

The splits mentioned above are related to the physiological function of the ABCs: *S*1 and *S*10 are iron transporters of *Streptomyces coelicolor *(*S*1) and *E. coli *(*S*10), *S*2 and *S*4 are related to the daunorubizin resistance of *Streptomyces peucetius *(*S*2) and *Rhizobium leguminosum *(*S*4), *S*9 and *S*11 transport amino acids in *E. coli *and *S*5 and *S*6 are ABCs from the MURA-RPON intergenic region of *E. coli *and from the NTRA/RPON 5' region of *Rhizobium meliloti*.

Finally, *S*13 is a phosphonates transporter of *E. coli*, whereas *S*8, *S*20, *S*21, and *S*22 are transporter of peptides: *S*8 is a dipeptide transporter of *Bacillus subtilis*, whereas *S*20, *S*21, and *S*22 are oligopeptide transporters of *Sinorhizobium meliloti*. Furthermore, the trees constructed for ⅅ*' *suggest that the common precursor of these five sequences differentiated into the phosphonates transporter *S*13 and the common precursor of the four peptide transporters *S*8, *S*20, *S*21, and *S*22. They also suggest that this precursor differentiated further into the dipeptide transporter *S*8 and the common precursor of the three oligopeptide transporters *S*20, *S*21, and *S*22 that evolved from this precursor by motif duplication which, in turn, differentiated further into *S*21 and the common precursor of *S*20 and *S*22 showing a stronger conservation of their N-terminus copy of the – **A **– **S **– **B **– motif than of their C-terminus copy. These parts of the sequences apparently were allowed to adapt to the new function more freely, suggesting that, after all, the class-5 alignments might, in spite of their lower DIALIGN score, be more appropriate than the class-3 alignments.

## 5 Summary and discussion

Phylogenetic tree reconstruction from sequence data can be a very difficult task that involves two major subproblems, namely (*a*) to construct a *multiple alignment *of the input sequences in such a way that biologically related parts of these sequences are assigned to each other and (*b*) to construct a *phylogenetic tree *based on such an alignment. If these subtasks are to be solved algorithmically, two types of questions are to be addressed: suitable *scoring functions *need to be defined assigning quantitative *confidence scores *to each possible multi-alignment or phylogenetic tree, respectively. These scoring functions should be defined in such a way that biologically reasonable alignments or trees are more likely to receiving higher scores than biologically meaningless ones. Given such a scoring scheme, the second problem is an *optimization problem*, namely to find alignments or trees, respectively, with optimal (or near-optimal) scores. Thus, phylogenetic tree reconstruction can be affected by two different types of errors. One kind of error reflects modeling problems such as unsuitable definitions of alignment scores or (dis)similarities for tree reconstruction. The other kind of errors are due to problems with finding the optimal alignment for a fixed alignment score and the optimal tree for a fixed (dis)similarity matrix. Both errors can, of course, produce instable alignments and phylogenies.

In this study, we restricted our attention to so-called *safe *branches of the phylogenies under investigation, i.e. to branches with bootstrap values above some threshold value. In view of their rather restrictive definition, it was natural to guess that that instabilities of safe branches indicate instabilities of the underlying alignments. And, indeed, we could relate distinct types of tree topologies to distinct alignment classes which were defined in terms of the relative position of the – **A **– **S **– **B **– motifs. Obviously, the threshold value of 5/6 that we used in our definition of safe branches is somewhat arbitrary. But, for the sequence set considered in this study, it worked well: the different types of tree topologies that we observed could be related to distinct classes of sequence alignments. For smaller threshold values, it is quite likely that more safe branches will lead to more tree-topology types demanding the consideration of more subtle differences between the corresponding alignments.

Furthermore, we could show that preventing certain pairs of sequence sites, or whole gap-free sequence segments, from being incorporated into a DIALIGN alignment by using the *xfr *option, resulted in the perfectly stable and biologically meaningful class-3 alignment which, in addition, had always a higher score than the other alignments. It is worth noting that this is no proof that the class-3 alignment represents the optimal alignment of our sequence set with respect to the DIALIGN score – there could be distinct alignments with even higher scores – and even less that it is the "biologically correct" alignment. But it allowed us to avoid alignments that are obviously suboptimal, and we could show that these class-3 alignments produce stable tree topologies. For lack of appropriate scoring parameters, a similar analysis could not be performed for CLUSTAL W. But we could demonstrate that small variations of the guide tree can result in rather different alignments and definitively distinct tree topologies. This might be a good explanation for the instabilities produced by CLUSTAL W for our sequence set.

The principal reason for the observed instabilities is probably the repetitive structure of some of the sequences under consideration. It is well known that repeats cause serious problems for all alignment algorithms [12]. Here, both subtasks in sequence alignment are concerned: it is possible that, due to repeats, biologically meaningful alignments receive lower scores than alternative, yet biologically unreasonable alignments. In addition, repeats can prevent optimization algorithms from finding (near-)optimal alignments. Both types of errors have been observed, for example, for the hox-gene cluster, see [26] for more details.

In this study, we obtained stable tree topologies after constructing new repeat-free sequences from our sequences using the sc option of DIALIGN. Using these new sequences, DIALIGN produced stable alignments whereas CLUSTAL W still produced unstable alignments, yet essentially stable phylogenies. An explanation for this observation (that unstable alignments can result in stable phylogenies) is perhaps that the threshold value 5/6 in the definition of safe branches is too high to reflect instabilities of the trees. Hence, in this case, one should actually use a lower threshold value, redo all analyses, and check whether such a refinement allows to find instabilities of the trees and to trace them back to the observed alignment instabilities. In our study, only a small set of ABCs was analysed. But, as will be shown in a forthcoming article (H. Wagner and N. Diaz, *in preparation*), analogous analyses are also required in the phylogenetic investigation of larger sets of ABCs – in particular, since quite a few ABCs have repetitive structure. Note that complete ABCs are composed of three kinds of domains, the ATPases, the transmembrane domains, and the periplasmatic binding domain. In the present study, we only considered repeats within the ATPases (see Section 3.1), but repeats can also occur in other domains.

Note that the requirement of stability is only a *necessary *but not a *sufficient *condition for the reliability of the output of an alignment or tree-reconstruction program. It does not *guarantee *its "correctness", and the same holds, of course, for score-optimal results: Even if one could show that a certain alignment has optimal score, that would not imply that it is also biologically correct since the underlying scoring function may assign optimal *numerical *scores to *biologically *wrong alignments. In general, a (near-)optimal alignment score only implies that the corresponding alignment might be a noteworthy approximation to biological reality. In fact, it is not *possible *to define scoring functions for sequence alignment in such a way that score-optimal alignments *necessarily *coincide with biologically correct ones. For real-world sequences, common ancestry and common function and structure do not always correspond to similarity at the primary-sequence level. This is a *fundamental *limitation for *all *automated alignment approaches. To overcome these limitations, *semi-automatic *alignment methods have been proposed where expert knowledge can be used to guide the alignment procedure. The *exclude fragments (xfr) *option introduced in this article offers one way to support such semi-automatic approaches. An even stronger option is the previously introduced *anchored alignment *option where the program is *forced *to align certain user-defined parts of the sequences to each other, see for example [27].

Stability analysis in phylogenetic studies is helpful to learn in more detail which alignments and (dis)similarities may be better descriptions of various aspects of sequence evolution. E.g., if phylogenetic analyses with different tools consistently lead to the same stable results, then there is at least a good chance that these results reflect the correct branching pattern of the evolution of the sequences under consideration. For similar reasons, we recently introduced a tool that compares distinct multi-alignments of the same sequence data set [25]. Such coincidences between safe branches of different trees were investigated in Section **4.5**. These safe branches were shown to separate ABCs with identical function from the rest where the function of an ABC was defined by the transported substrate. Thus, it can be assumed that such branches of phylogenetic trees can indeed be used for functional predictions as discussed in the introduction.

Note that, in our analyses, ABCs from different organisms with identical functions were grouped together, but not ABCs from the same organism with different functions. As noted already in [30], this suggests that the segregation of the ABCs according to their function is older than their segregation according to the evolution of their respective organisms. Additionally or alternatively, horizontal gene transfer – well established in the bacterial world – could also contribute to this phenomenon.

From our analyses, we may also postulate that the phosphonate transporters and the dipeptide and oligopeptides transporters have a common ancestor; yet, in contrast to the dipeptide transporters, the oligopepetide transporters have a repetitive structure. However, the data set here is too small to unravel their history in detail. In a forthcoming article, these aspects of sequence evolution will be considered in more detail (Wagner and Diaz, *in preparation*).
